# Thioridazine Induces Increase in Expression of the Pyruvate Transporter *MPC*1 Associated with Immune Infiltration in Malignant Tumors

**DOI:** 10.1134/S001249662460060X

**Published:** 2025-01-31

**Authors:** E. A. Bogomolova, M. M. Murashko, E. M. Stasevich, A. N. Uvarova, E. A. Zheremyan, K. V. Korneev, D. V. Kuprash, D. E. Demin

**Affiliations:** 1 Laboratory of Intracellular Signaling in Health and Disease, Engelhardt Institute of Molecular Biology, Russian Academy of Sciences, 119991 Moscow, Russia; 2 Center for Precision Genome Editing and Genetic Technologies for Biomedicine, Engelhardt Institute of Molecular Biology, Russian Academy of Sciences, 119991 Moscow, Russia; 3 Department of Molecular and Biological Physics, Moscow Institute of Physics and Technology, 141701 Dolgoprudny, Moscow oblast Russia

**Keywords:** non-small cell lung cancer, *MPC*1, thioridazine, schizophrenia, immune infiltration

## Abstract

The *MPC*1 gene is involved in the transport of pyruvate into mitochondria, playing an important role in metabolic processes. Recently, it has been reported that higher *MPC*1 expression correlates with an increased number of immune cells in human cervical and lung cancers, indicating an enhanced antitumor immune response. Reduced *MPC*1 levels in gastric tumors are associated with a more severe disease course. Correlational analysis of the *MPC*1 gene in human lung, hippocampus and frontal cortex tissue samples based on data from the GTEx database revealed associations of this gene with schizophrenia, non-small cell lung cancer, and immune diseases. Our experiments showed that the mRNA level of the *MPC*1 gene in the non-small cell lung cancer cell line A549 increases 5-fold under the influence of the schizophrenia neuroleptic thioridazine. The observed elevation of *MPC*1 level may cause tumor infiltration by immune cells, complementing the previously reported data indicating the ability of thioridazine to slow cell growth, induce apoptosis and reduce the ability of cells to migrate.

## INTRODUCTION

Non-small cell lung cancer (NSCLC) accounts for about 85% of all cases of malignant lung tumors [1]. The immunologic environment of the tumor plays a key role in cancer development and progression as well as response to therapy. The introduction of immune checkpoint inhibitors, such as CTLA-4 and PD-1/PD-L1 blockers, has been a breakthrough in the treatment of non-small cell lung cancer. However, many patients do not respond to treatment or show disease progression. This highlights the need to develop new biomarkers and therapeutic approaches based on the interaction between the tumor and the immune system [2].

The *MPC*1 gene plays a key role in the transport of pyruvate across the inner mitochondrial membrane, ensuring its participation in oxidative phosphorylation and energy metabolism of the cell. Recent studies have shown that increased expression levels of the *MPC*1 gene are associated with the infiltration of immune cells into lung cancer tumor tissue. In particular, there is a correlation between *MPC*1 level and the number of follicular T-helper cells and eosinophils, which may be related to the effectiveness of the antitumor immune response [3]. In addition, correlation of *MPC*1 expression with immune infiltration is found in cervical cancer [4]. Reduced *MPC*1 levels are associated with poor prognosis and tumor progression in gastric cancer [5].

The mitochondrial pyruvate transporter complex (MPC) is known to consist of MPC1 and MPC2 subunits, and loss of either of them leads to dysfunction of the entire complex. At the same time, the single nucleotide polymorphism rs10489202 in an intron of the *MPC*2 gene is associated with schizophrenia in East Asian populations [6].

In recent years, the antipsychotic drug thioridazine, used in the treatment of schizophrenia, has attracted the attention of researchers as a potential antitumor agent, especially against various cancers, including lung cancer [7]. The ability of thioridazine to influence inflammation by blocking IKKβ kinase, which in turn prevents the activation of the transcription factor NF-κB, has been shown [8]. This drug is able to induce apoptosis, inhibit cancer cell proliferation and migration, and selectively destroy cancer stem cells, making it an interesting candidate to target recurrent and resistant forms of cancer [9]. The effects of thioridazine against cancer cells are related to the activation of mitochondrial apoptosis through inhibition of fatty acid oxidation. It causes a decrease in mitochondrial membrane potential, caspase-9 activation, increased Bax protein levels and decreased Bcl-2 levels, leading to cell death [7]. Thioridazine administration is associated with a decreased risk of gastric cancer [10].

In this study, we analyzed the mRNA expression of *MPC1* gene in human lung tissue samples based on the GTEx database. Based on the data available in the literature on the effect of thioridazine on mitochondrial apoptosis, we studied the effect of thioridazine on *MPC1* gene expression in the non-small cell lung cancer cell line A549.

## MATERIALS AND METHODS

**Cells lines.** The A549 non-small cell lung cancer cell line was used in the study. Cells were cultured in DMEM medium containing 4.5 g/L glucose (PanEco), supplemented with a mixture of antibiotics, including penicillin (100 U/mL) and streptomycin (100 µg/mL) (PanEco), 1% non-essential amino acids solution (PanEco), 10 mM HEPES (GIBCO), and 10% fetal bovine serum (Biosera). Thioridazine (PHARMACEUTICAL WORKS JELFA S.A.) was added to the cells at concentrations of 10, 20, 30 or 40 µM, while the control samples received an equivalent volume of culture medium.

**Correlational analysis of the** ***MPC1*** **gene.** To identify the biological processes involving the *MPC*1 gene, a correlation analysis was performed as described in [11]. Briefly, RNA sequencing data from normal human tissues were used from the GTEx database:578 lung samples, 197 hippocampus samples, and 209 frontal cortex samples (BA9). The Spearman correlation of gene expression for all genes with *MPC*1 expression was calculated for these tissues. The top 1000 genes with the highest absolute correlation coefficients were then used for gene set enrichment analysis using Metascape and EnrichR.

**RNA isolation and quantitative polymerase chain reaction.** Total RNA was isolated from cells 24 h after transfection using ExtractRNA reagent (Eurogen) according to the protocol recommended in the kit. cDNA from total RNA was prepared with the MMLV RT kit (Eurogen) using oligo-dT primers and random nucleotide primers in a 1-to-1 ratio as described in [12]. Expression analysis was performed by quantitative polymerase chain reaction (qPCR) on a CFX96 Touch real-time PCR instrument (Bio-Rad Laboratories) using qPCRmix-HS SYBR reagents (Eurogen) and specific primers: for *MPC*1 gene—GGACTATGTCCGAAGCAAGG; AAATGTCATCCGCCCACTGA. Normalization was performed for the beta-actin ACTB gene—ACTGGGGACGACGACATGGAGAGAAA; GGCGTACAGGGATAGGATAGCACAG. The cDNA obtained from 100 ng of isolated RNA was used per triplicate quantitative PCR.

## RESULTS AND DISCUSSION

### MPC1 mRNA Expression in Human Lung Tissue Correlates with Genes from Groups Associated with Schizophrenia, Lung Cancer, and Immunopathologies

As a result of the gene set enrichment analysis based on the list of one thousand genes most strongly correlated with *MPC*1 in lung and brain tissues (hippocampus and frontal cortex BA9), the biological processes in which the *MPC*1 gene may be involved were identified.

**Fig. 1.**
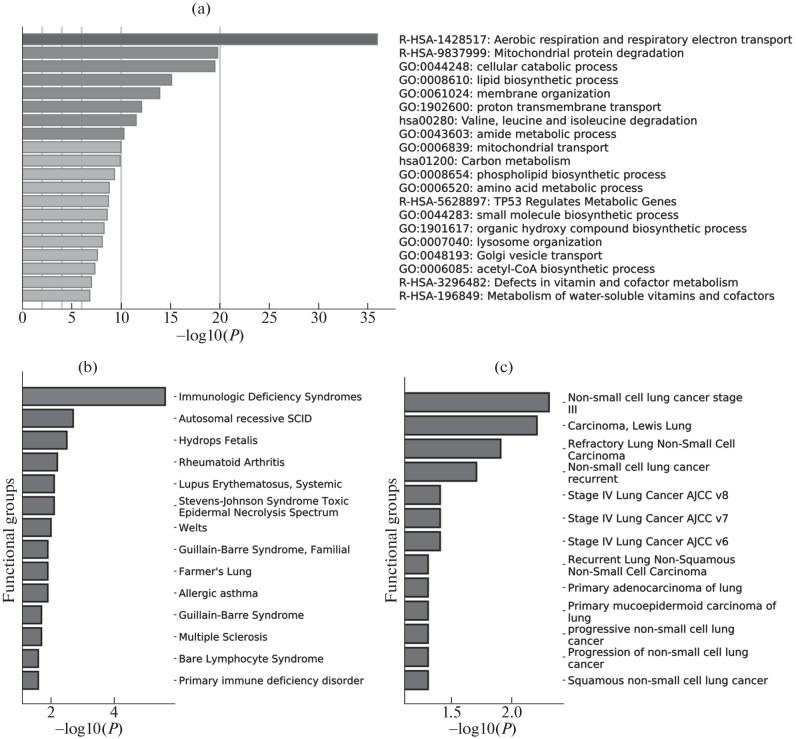
Genes correlating with *MPC*1 in lung tissues enrich functional groups associated with metabolic processes (a), immunopathologies (b), and lung cancer (c). The figure shows groups of genes obtained as a result of correlation analysis of mRNA *MPC*1 expression in human lung tissue samples. (a) The strongest enrichment of functional groups obtained using the Metascape service. Enrichment of groups of genes from the DisGeNET database related to immune diseases (b) and lung cancer (c).

In normal human lung tissues, processes potentially associated with *MPC*1 include mitochondrial transport, amide metabolism, valine, leucine, and isoleucine degradation, proton transmembrane transport, membrane organization, lipid biosynthetic process, cellular catabolic process, mitochondrial protein degradation, and aerobic respiration and respiratory electron transport (Fig. 1a).

In addition to understanding common processes, the association with biological processes from the DisGeNet database that are associated with diseases is also of interest. We identified a variety of potential associations between the *MPC*1 gene and groups, and selected groups that are associated with immune diseases and lung cancer to show in the figure. Figure 1b displays the groups associated with immune diseases: immunodeficiencies, autoimmune diseases, neurological disorders, and allergic reactions. These results suggest a possible link between *MPC*1 and immune regulation and the pathogenesis of various immunologic conditions.

Figure 1b displays the groups associated with lung cancer: non-small cell lung cancer, its advanced and recurrent forms, and stages III and IV. Tumor types such as primary adenocarcinoma of the lung, squamous cell non-small cell lung cancer, and various carcinomas of the lung were also identified. The observed associations are in good agreement with the data on the prognostic significance of *MPC*1 in lung cancer [13] and its possible role in pathogenesis [14].

The formation of the MPC1 and MPC2 protein complex, as well as the association of MPC2 with schizophrenia, suggests in turn that the *MPC*1 gene is related to this psychiatric disorder. Enrichment analysis of functional gene groups in brain tissues associated with schizophrenia found that for hippocampal samples, schizophrenia is in the top 3, and in frontal lobe (BA9) samples in a list of the 15 most enriched groups.

The results of the correlation analysis emphasize the role of *MPC*1 in metabolic processes and maintenance of lung tissue homeostasis, and suggest an association with schizophrenia in brain tissue. Thus, the results obtained in conjunction with the literature indicate that *MPC*1 can potentially be considered as a biomarker associated with the development of lung cancer, immunopathologies and schizophrenia.

### Thioridazine Increases the mRNA Expression Level of the MPC1 Gene

**Fig. 2.**
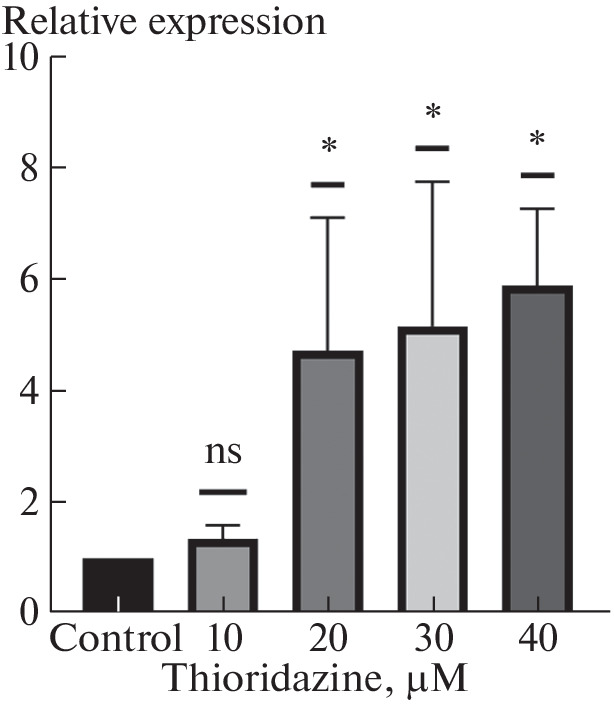
Effect of thioridazine on the MPC1 mRNA expression. QPCR detection of MPC1 expression with different concentrations of thioridazine. * means p-value lower than 0.05 according to the one-sample Wilcoxon test.

It was previously shown that the antipsychotic thioridazine used in schizophrenia slows growth and reduces the ability of non-small cell lung cancer cells to migrate, and at higher concentrations leads to apoptosis [15]. Our results showed that at a thioridazine concentration of 10 μM, no significant increase in *MPC*1 gene mRNA expression was observed. At a concentration of 20 μM, a significant increase of 5 ± 2-fold was already seen, further at concentrations of 30  and 40 μM, a marked increase in *MPC1* mRNA levels of 5 ± 3 and 5.9 ± 1.4-fold, respectively, was also found (Fig. 2).

Therapeutic dosages of thioridazine in the treatment of various psychiatric disorders such as schizophrenia are up to 400 mg/day, with concentrations on the order of 10 μM observed in human serum at 400 mg/day dosage [16, 17]. Therefore, the relevance of this cell model to the therapeutic use of thioridazine requires a separate investigation. However, in clinical studies, the required concentrations of the drug are often several times lower than those used in vitro [18]. It should be noted that previous studies have found a correlation between increased *MPC*1 expression and infiltration of lung tumors by follicular T-helper cells and eosinophils [8], and infiltration of non-small cell lung cancer by follicular T-helper cells is associated with a good clinical prognosis [19]. It can be assumed that the increase in *MPC*1 during thioridazine exposure may participate in the attraction of these types of immune cells to the tumor.

## References

[1] Minna, J.D., Roth, J.A., and Gazdar, A.F., Focus on lung cancer, *Cancer Cell*, 2002, vol. 1, no. 1, pp. 49–52. 10.1016/S1535-6108(02)00027-2 12086887 10.1016/s1535-6108(02)00027-2

[2] Saigi, M., Alburquerque-Bejar, J.J., and Sanchez-Cespedes, M., Determinants of immunological evasion and immunocheckpoint inhibition response in non-small cell lung cancer: the genetic front, *Oncogene*, 2019, vol. 38, no. 31, pp. 5921–5932. 10.1038/s41388-019-0855-x 31253869 10.1038/s41388-019-0855-x

[3] Li, F., Huang, C., Zhou, M., et al., MPC1 is down-regulated in lung cancer and associated with immune infiltration, *Proceedings of the Fourth International Conference on Biomedicine and Bioinformatics Engineering*, Piccaluga, P.P., El-Hashash, A., and Guo, X., Eds., 2024, vol. 27. 10.1117/12.3044194

[4] Li, M., Xu, T., Yang, R., Wang, X., Zhang, J., and Wu, S., Exploring MPC1 as a potential ferroptosis-linked biomarker in the cervical cancer tumor microenvironment: a comprehensive analysis, *BMC Cancer*, 2024, vol. 24, no. 1, p. 1258. 10.1186/s12885-024-12622-x 39390460 10.1186/s12885-024-12622-x PMC11465577

[5] Zhou, X., Xiong, Z.J., Xiao, S.M., et al. Overexpression of MPC1 inhibits the proliferation, migration, invasion, and stem cell-like properties of gastric cancer cells, *OncoTargets Ther*., 2017, vol. 10, pp. 5151–5163. 10.2147/OTT.S148681 10.2147/OTT.S148681 PMC5661476 29123413

[6] Yang, Y., Wang, L., Li, L., et al., Genetic association and meta-analysis of a schizophrenia GWAS variant rs10489202 in East Asian populations, *Transl. Psychiatry*, 2018, vol. 8, no. 1, pp. 1–11. 10.1038/s41398-018-0211-x 30087317 10.1038/s41398-018-0211-x PMC6081446

[7] Qian, G., Dai, L., and Yu, T., Thioridazine sensitizes cisplatin against chemoresistant human lung and ovary cancer cells, *DNA Cell Biol*., 2019, vol. 38, no. 7, pp. 718–724. 10.1089/dna.2019.4715 31188023 10.1089/dna.2019.4715

[8] Baig, M.S., Roy, A., Saqib, U., et al., Repurposing Thioridazine (TDZ) as an anti-inflammatory agent, *Sci. Rep*., 2018, vol. 8, no. 1, p. 12471. 10.1038/s41598-018-30763-5 30127400 10.1038/s41598-018-30763-5 PMC6102213

[9] Shen, J., Ma, B., Zhang, X., et al., Thioridazine has potent antitumor effects on lung cancer stem‑like cells, *Oncol. Lett*., 2017, vol. 13, no. 3, pp. 1563–1568. 10.3892/ol.2017.5651 28454291 10.3892/ol.2017.5651 PMC5403693

[10] Lianos, G.D., Alexiou, G.A., Rausei, S., Galani, V., Mitsis, M., and Kyritsis, A.P., Repurposing antipsychotic drugs for cancer treatment: current evidence and future perspectives, *Expert Rev. Anticancer Ther*., 2022. Accessed October 25, 2024. https://www.tandfonline.com/doi/abs/10.1080/14737140.2022.1987221 10.1080/14737140.2022.1987221 34612130

[11] Demin, D.E., Murashko, M.M., Uvarova, A.N., et al., Adversary of DNA integrity: A long non-coding RNA stimulates driver oncogenic chromosomal rearrangement in human thyroid cells, *Int. J. Cancer*, 2023, vol. 152, no. 7, pp. 1452–1462. 10.1002/ijc.34396 36510744 10.1002/ijc.34396

[12] Demin, D.E., Stasevich, E.M., Murashko, M.M., Tkachenko, E.A., Uvarova, A.N., and Schwartz, A.M., Full and D-Box-Deficient PTTG1 Isoforms: Effects on cell proliferation, *Mol. Biol.* (Moscow), 2022, vol. 56, no. 6, p. 1104. 10.31857/S0026898422060076 10.31857/S0026898422060076 36475495

[13] Zou, H., Yin, Y., Xiong, K., et al., Mitochondrial pyruvate carrier 1 as a novel prognostic biomarker in non-small cell lung cancer, *Technol. Cancer Res. Treat*., 2024, vol. 23, p. 15330338241282080. 10.1177/15330338241282080 10.1177/15330338241282080 PMC11452851 39360506

[14] Zou, H., Chen, Q., Zhang, A., et al., MPC1 deficiency accelerates lung adenocarcinoma progression through the STAT3 pathway, *Cell Death Dis.*, 2019, vol. 10, no. 3, p. 148. 10.1038/s41419-019-1324-8 30770798 10.1038/s41419-019-1324-8 PMC6377639

[15] Huang, W., Thioridazine promotes primary ciliogenesis in lung cancer cells through enhancing cell autophagy, *Int. J. Clin. Exp. Med*., 2017, vol. 10, pp. 13960–13969.

[16] Mårtensson, E. and Roos, B.E., Serum levels of thioridazine in psychiatric patients and healthy volunteers, *Eur. J. Clin. Pharmacol*., 1973, vol. 6, no. 3, pp. 181–186. 10.1007/BF00558283 4762056 10.1007/BF00558283

[17] Cohen, B.M., Lipinski, J.F., and Waternaux, C., A fixed dose study of the plasma concentration and clinical effects of thioridazine and its major metabolites. *Psychopharmacology*, 1989, vol. 97, no. 4, pp. 481–488. 10.1007/BF00439552 2498945 10.1007/BF00439552

[18] Groothuis, F.A., Heringa, M.B., Nicol, B., Hermens, J.L.M., Blaauboer, B.J., and Kramer, N.I., Dose metric considerations in *in vitro* assays to improve quantitative *in vitro*-*in vivo* dose extrapolations, *Toxicology*, 2015, vol. 332, pp. 30–40. 10.1016/j.tox.2013.08.012 23978460 10.1016/j.tox.2013.08.012

[19] Ma, Q.Y., Huang, D.Y., Zhang, H.J., Chen, J., Miller, W., and Chen, X.F., Function of follicular helper T cell is impaired and correlates with survival time in non-small cell lung cancer, *Int. Immunopharmacol*., 2016, vol. 41, pp. 1–7. 10.1016/j.intimp.2016.10.014 27788370 10.1016/j.intimp.2016.10.014

